# Prognostic impact of vascular invasion and standardization of its evaluation in stage I non-small cell lung cancer

**DOI:** 10.1186/s13000-015-0249-5

**Published:** 2015-04-02

**Authors:** Rurika Hamanaka, Tomoyuki Yokose, Yuji Sakuma, Masahiro Tsuboi, Hiroyuki Ito, Haruhiko Nakayama, Kouzo Yamada, Ryota Masuda, Masayuki Iwazaki

**Affiliations:** Department of Thoracic Oncology, Kanagawa Cancer Center Hospital, Yokohama, Japan; Division of General Thoracic Surgery, Department of Surgery, Tokai University School of Medicine, 143 Shimokasuya, Isehara, Kanagawa 259-1193 Japan; Department of Pathology, Kanagawa Cancer Center Hospital, 2-3-2 Nakao, Asahi-Ku, Yokohama, Kanagawa 241-8515 Japan; Division of Thoracic Surgery, Respiratory Disease Center Yokohama City University Medical Center, 4-57 Urafune, Minami-Ku, Yokohama, Kanagawa 232-0024 Japan

**Keywords:** Non-small cell carcinoma, Stage I, Blood vessel invasion, Lymphatic vessel invasion, Hematoxylin-eosin staining, Elastica van Gieson staining, Podoplanin, D2-40

## Abstract

**Background:**

Patients with pathologic stage (p-Stage) IA non-small cell lung cancer (NSCLC) have a good survival rate because of possible curative resection. However, up to 10% of these patients relapse postoperatively. To identify unfavorable prognostic factors, we retrospectively analyzed the clinicopathological features of p-Stage IA disease, focusing on vascular invasion.

**Methods:**

Of 467 patients with p-Stage I NSCLC, 335 were diagnosed with p-Stage IA or IB disease based on a lesion size ≤3 cm and the presence of pleural invasion (PL). Univariate and multivariate analyses of recurrence-free survival (RFS) were performed with age, sex, PL, and vascular invasion (blood vessel invasion [v] and lymphatic vessel invasion [ly]) as variables. To examine vascular invasion, hematoxylin-eosin (HE), Elastica van Gieson staining, and immunostaining with anti-podoplanin antibody were performed. The presence or absence of v and ly was recorded; the number of involved vessels was counted. Survival rates were obtained using the Kaplan–Meier method and log-rank test. Multivariate analyses were performed using the Cox proportional hazards model.

**Results:**

RFS differed significantly between patients with no or one involved blood vessel (0 v or 1 v) and those with ≥2 involved vessels (≥2 v). Similarly, RFS differed significantly between patients with no lymphatic vessel involvement (0 ly) and those with one involved lymphatic vessel (1 ly). Thus, BVI(+) and BVI(−) were defined as ≥2 v and 0 v + 1 v, and LVI(+) and LVI(−) as ≥1 ly and 0 ly, respectively. BVI and LVI together represented tumor vessel invasion (TVI). On multivariate analyses, PL and TVI were independently associated with recurrence. Additionally, patients with p-Stage IA TVI(+) disease had a comparable recurrence rate to those with p-Stage IB disease.

**Conclusions:**

Similar to PL, TVI is an important factor increasing the likelihood of recurrence. As HE staining alone is insufficient for evaluating vascular invasion, specific staining is necessary. Moreover, patients with p-Stage IA TVI(+) disease had a recurrence rate comparable to those with p-Stage IB disease; therefore, further studies should aim to elucidate whether patients with p-Stage IA TVI(+) disease should be administered postoperative chemotherapy similar to that received by p-Stage IB patients.

**Virtual Slides:**

The virtual slide(s) for this article can be found here: http://www.diagnosticpathology.diagnomx.eu/vs/5213064891369688

## Background

Postoperative chemotherapy is currently administered to patients with pathological stage (p-Stage) IB cancer, as the efficacy of postoperative adjuvant chemotherapy in these patients has been proven in several global studies [1-3], and postoperative oral tegafur-uracil has proved efficacious in a group of Japanese patients [4,5]. At present, patients with p-Stage IA cancer often only receive follow-up care after surgery because their outcome is generally favorable without postoperative chemotherapy. Therefore, it is difficult to determine the efficacy of postoperative chemotherapy in these patients. In addition, chemotherapy including tegafur-uracil is not currently administered to patients with p-Stage IA disease, because of the observed adverse effects. However, identification of patients with p-Stage IA disease who have a high risk of recurrence may result in further improvement in the outcome of these patients.

Pleural invasion (PL) is a factor used in the tumor-node-metastasis (TNM) classification, and its importance as a poor prognostic factor has been demonstrated in several studies [6-11]. In the 7th edition of the Union for International Cancer Control TNM Classification, published in 2009, PL was reappraised as a pathological factor that distinguishes p-Stage IA from p-Stage IB disease. While invasion at the pleural surface denoted p-Stage IB in the previous editions, invasion beyond the pleural elastic layer became a criterion for determining p-Stage IB in the 7th edition. Travis et al. [12,13] recommended the use of elastic fiber staining to improve the accuracy of its determination.

Despite many previously published studies reporting that vascular invasion, comprising blood vessel invasion (v) and lymphatic vessel invasion (ly), was a strong independent prognostic factor for recurrence in patients with p-Stage I primary non-small cell lung cancer (NSCLC) lesions ≤3 cm in diameter [10,14-23], it was not considered a factor in the new TNM classification. This may explain why there is no standard pathological assessment method for vascular invasion.

Accordingly, we assessed vascular invasion in patients with p-Stage I NSCLC lesions ≤3 cm diameter, using hematoxylin-eosin (HE), Elastica van Gieson (EvG), and D2-40 antibody immunohistological staining to examine whether vascular invasion can be assessed using HE staining alone. In addition, we evaluated whether vascular invasion was a useful prognostic factor in patients with p-Stage I NSCLC lesions ≤3 cm in diameter. We assessed the impact of vascular invasion by using the number of invaded vascular channels as a prognostic factor and compared its prognostic accuracy with the presence or absence of PL. Furthermore, we investigated whether v and ly are independent prognostic factors for recurrence in patients with p-Stage I NSCLC by comparing them with other factors, and examined whether differences in recurrence rates according to vascular invasion are useful in reconsidering the application of additional postoperative treatment.

## Methods

### Patients

A total of 467 patients with p-Stage I NSCLC who underwent a radical lobectomy during the period between January 2000 and December 2005 at Kanagawa Cancer Center Hospital were evaluated. Totally, 335 patients were diagnosed with p-Stage IA or IB disease based on a lesion size ≤3 cm in diameter and the presence or absence of PL (observation period: 8–4010 days; median: 2292 days); 132 patients were diagnosed with p-Stage IB disease with a tumor diameter of 3–5 cm, irrespective of PL (observation period: 34–3939 days; median: 1932.5 days). All patients were consecutively enrolled, and none were excluded. The patients did not receive preoperative treatment or additional postoperative treatment during the recurrence-free follow-up period. The study was performed according to the requirements of the Institutional Review Board of Kanagawa Cancer Center.

### Study design

1. A study on the significance of vascular invasion in patients with p-Stage I NSCLC lesions ≤3 cm in diameter was performed in 335 patients with p-Stage IA or IB cancer based on the presence of PL.
  i) Objective determination of vascular invasion
    A comparative study was performed focusing on v, ly, and PL, which should be regarded as factors causing the reclassification of T1 lung cancer lesions ≤3 cm in diameter as T2a cancer in the 7th TNM classification. Univariate and multivariate analyses were performed for recurrence-free survival (RFS) periods with age, sex, PL, and vascular invasion as variables.
    To examine vascular invasion, samples for HE and EvG staining and immunostaining with anti-podoplanin antibody (clone D2-40) were newly prepared from whole paraffin blocks of the largest tumor sections. The stained samples were evaluated in the aforementioned order to permit comparison of the findings of the respective stained samples. First, the presence or absence of v and ly in the largest sections was determined on HE staining alone. The criterion for the presence or absence of invasion was destruction of the vascular wall or further invasion into the vascular lumen. Later, the presence or absence of v or ly was determined by using EvG or D2-40 staining, respectively. The HE staining assessment results remained unchanged after microscopic examinations of the EvG- and D2-40-stained samples. The entire surface of each sample was observed at × 10 magnification or up to × 40 in cases of unclear findings. Each sample was independently examined microscopically by two researchers (R.H. and T.Y.), and controversial cases were evaluated using a multi-headed microscope until they reached a consensus. The presence of PL was determined by the detection of tumor cells on the pleural surface on HE staining, or the observation of invasion beyond the pleural elastic layer on EvG staining.
  ii) Assessment of the number of invaded vessels
    The numbers of invaded blood and lymphatic vessels were counted in patients with lung cancer lesions ≤3 cm in diameter that were positive for vascular invasion on EvG or D2-40 staining. The entire surface of each sample was observed at × 10 magnification or up to × 40 in cases of unclear findings. Each case was independently examined microscopically by two researchers, and controversial cases were discussed until they reached an agreement.
2. A study on the relationship between vascular invasion, and p-Stages IA and IB included patients with p-Stage IB disease and a pathological tumor diameter of >3 cm. They were evaluated for the presence or absence of v or ly and the number of invaded vessels on EvG staining or D2-40, respectively. The protocol used was similar to that mentioned previously. p-Stage I was subdivided into p-Stage IA and IB according to the presence or absence of vascular invasion and the number of invaded vessels, and the results were compared between the two groups.

### Observation period and statistical analysis

All analyses were performed for the observation period (from the date of surgery to recurrence or the last known recurrence-free date). The recurrence date was defined as the date when recurrence was observed by using imaging studies. Statistical analyses were performed using the Statistical Package for the Social Sciences (Dr SPSS II for Windows, Tokyo, Japan, released 2001) software. Survival rates were calculated using the Kaplan-Meier method and analyzed using the log-rank test. Multivariate analyses were performed using the Cox proportional hazards model. Differences were considered significant when p < 0.05.

## Results

### Patient characteristics

Table 1 shows the patients’ characteristics. The age range for the 335 patients with p-Stage IA or IB disease based on PL was 29–86 years (mean, 64 years; median, 65 years). There were 164 male and 171 female patients. A total of 170 patients were smokers, and 165 were non-smokers. Two hundred and ninety-nine patients were diagnosed with p-Stage IA disease and 36 with p-Stage IB disease owing to the presence of PL. Two hundred and eighty-eight patients had adenocarcinoma, 24 had squamous cell carcinoma, and 23 had other cancers (e.g., large cell neuroendocrine carcinoma and large cell carcinoma). Recurrence was observed in 28 patients.

**Table 1:** **Patients’ characteristics**

		**All**	**≤3 cm (IA or IB [PL])**	**3–5 cm (IB)**
Number of patients		467	335	132
Age range	Years	29–88	29–86	46–88
	(mean/median)	(65.4/66)	(64/65)	(68.6/69.5)
Sex	Male	234	164	70
	Female	233	171	62
Smoking status	Smoker	247	170	77
	Non-smoker	220	165	55
p-Stage	IA	299	299	0
	IB	168	36	132
Histological differentiation	Adenocarcinoma	387	288	99
	Squamous cell	47	24	23
	carcinoma			
	Others	33	23	10
Recurrence		51	28	23
Deaths	Present	81	45	36
	Absent	386	290	96
(Cause of death)	(Lung cancer)	(37)	(18)	(19)

The age range of the 132 patients with p-Stage IB NSCLC lesions 3–5 cm in diameter was 46–88 years (mean, 68.6 years; median, 69.5 years). There were 70 male and 62 female patients. Seventy-seven patients were smokers, and 55 were non-smokers. Ninety-nine patients had adenocarcinoma, 23 had squamous cell carcinoma, and 10 had other cancers (e.g., large cell neuroendocrine carcinoma and large cell carcinoma). Recurrence was observed in 23 patients.

### Significance of vascular invasion in patients with p-Stage I NSCLC lesions ≤3 cm in diameter

**Figure 1:**
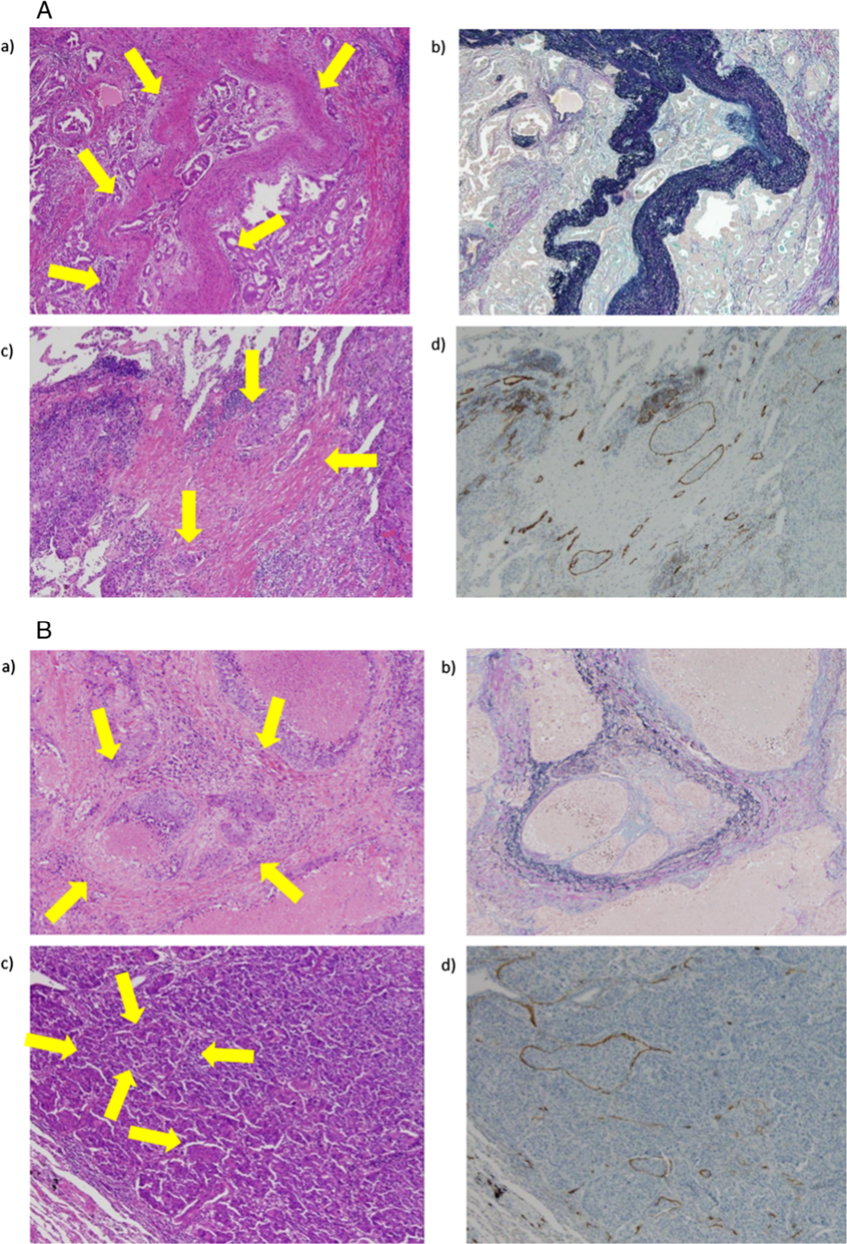
**Detection of vascular invasion by hematoxylin-eosin (HE) staining and other staining methods. A**: Representative case of vascular invasion by HE staining and confirmed using other staining methods. **a)** HE staining for the diagnosis of blood vessel invasion (v). **b)** Elastica van Gieson staining to confirm v in the same patient. **c)** HE staining identified multiple instances of lymphatic vessel invasion (ly). **d)** D2-40 staining to confirm ly in the same patient. **B**: Representative case of false-negative vascular invasion identified on HE staining. **a)** v could not be determined on HE staining. **b)** Elastica van Gieson staining showed v in the same patient. **c)** Lymphatic vessel invasion (ly) could not be determined on HE staining. **d)** D2-40 staining showed multiple instances of ly in the same patient.

i) Objective determination of vascular invasion
  Figure 1A contains representative images of vascular invasion on HE and other staining methods, and Figure 1B shows representative cases of false-negative vascular invasion identified on HE staining. Of the 30 patients diagnosed with v on HE staining alone, 2 were found to be false positive on EvG staining. Of the 305 patients diagnosed as not having v on HE staining, v was detected in 33 on EvG staining (false negatives; Table 2). Similarly, of 15 patients showing ly on HE staining alone, 3 were found not to have ly on D2-40 staining. Conversely, of the 320 patients diagnosed as not having ly on HE staining, ly was detected in 39 on D2-40 staining (Table 3).

**Table 2:** **Hematoxylin-eosin (HE) and Elastica van Gieson (EvG) staining to detect blood vessel invasion (v) frequency**

		**EvG**	
		**v(+)**	**v(−)**
HE	v(+)	28	2
	v(**−**)	33	272

**Table 3:** **Comparison of frequency of lymphatic vessel invasion (ly) with hematoxylin-eosin (HE) versus D2-40 antibody staining**

		**D2-40**	
		**ly(+)**	**ly(−)**
HE	ly(+)	12	3
	ly(**−**)	39	281

Table 4 shows the sensitivity and specificity of HE staining of v and ly versus EvG and D2-40 staining, which are recognized as gold standards for v and ly, respectively. Detection frequencies of both v and ly on HE staining showed high specificity, but very low sensitivity. Therefore, we decided to base v on the results of EvG staining and ly on the results of D2-40 staining.

**Table 4:** **Sensitivity and specificity of hematoxylin-eosin staining for blood vessel invasion (v) and lymphatic vessel invasion (ly)**

	**v**	**ly**
Sensitivity	46%	23.5%
Specificity	99.3%	98.9%

ii) Assessment according to the number of invaded vessels
  The 335 patients with tumors ≤3 cm in diameter were classified according to the number of invaded vessels. RFS was determined by dividing patients according to the presence and absence of v and ly following EvG and D2-40 staining, respectively. The probability of recurrence differed significantly between patients with a single involved vessel (1 v) compared to those with two or more involved vessels (≥2 v; p = 0.0131), whereas the risk of recurrence did not differ significantly between patients with no vessel involvement (0 v) and those in the 1 v group (p = 0.3673; Figures 2A, B). Similarly, the probability of recurrence differed significantly between patients with no lymphatic vessel invasion (0 ly) compared to those with invasion of one lymphatic vessel (1 ly; p = 0.0009), whereas the risk of recurrence was not significantly different between patients with 1 ly and those with invasion of two or more lymphatic vessels (≥2 ly; Figures 3A, B).

**Figure 2:**
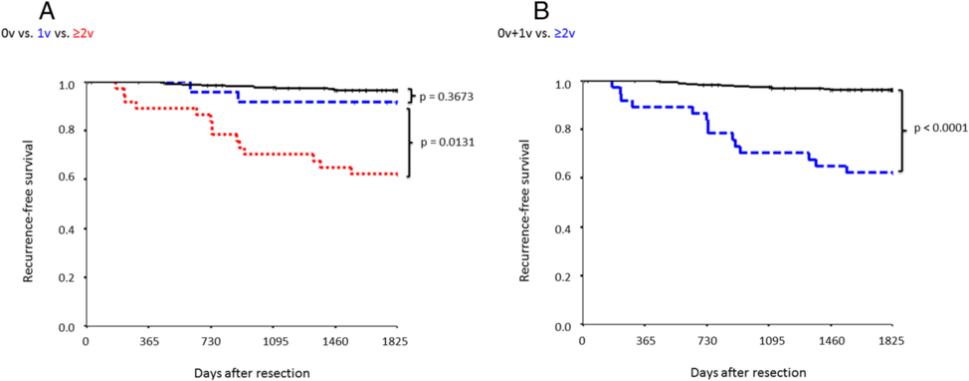
**Recurrence-free survival according to the number of invaded blood vessels. A) Comparison of 0 v (n = 274), 1v (n = 24), and ≥2 v (n = 37) cases. B)** Comparison between 0 v + 1 v (n = 298) and ≥2 v (n = 37) cases.

**Figure 3:**
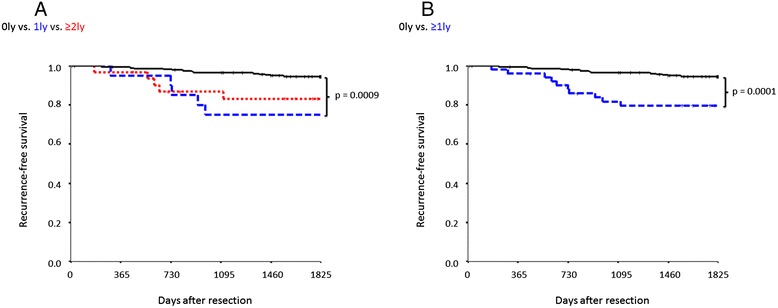
**Recurrence-free survival according to the number of invaded lymphatic vessels. A) Comparison of 0 ly (n = 284), 1 ly (n = 20), and ≥2 ly (n = 31) cases. B)** Comparison between 0 ly (n = 284) and ≥1 ly (n = 51) cases.

From this point, v was classified as 0 + 1 v or ≥2 v, denoting BVI(−) and BVI(+) invasion, respectively; ly was classified as 0 ly or ≥1 ly, denoting LVI(−) and LVI(+) invasion, respectively. Furthermore, BVI and LVI together represented tumor vessel invasion (TVI).

iii) Analysis of RFS in NSCLC patients with lesions ≤3 cm in diameter
  PL was observed in 36 of 335 patients with NSCLC lesions ≤3 cm in diameter. Univariate analyses revealed significant differences in the duration of RFS according to the presence or absence of PL, BVI, and LVI (PL, p < 0.0001; BVI, p < 0.0001; LVI, p = 0.0001; Table 5). Multivariate analyses revealed that all of the aforementioned factors were independently involved in recurrence (PL, hazard ratio [HR] = 2.799; BVI, HR = 5.669; LVI, HR = 2.335; Table 6). In addition, multivariate analyses also revealed that TVI was predictive of recurrence (HR = 6.946; Table 7).

**Table 5:** **Recurrence-free survival and clinicopathological characteristics**

**Variables**		**No.**	**RFP (%)**	**Univariate P value**
All		335	92.3	
Age (years)	≥65	178	94.4	0.3224
	<65	157	90.1	
Sex	Male	164	91.2	0.3002
	Female	171	93.5	
Smoking status	Smoker	165	94.5	0.1013
	Non-smoker	170	90.4	
PL	(−)	299	94.5	<0.0001
	(+)	36	74.6	
BVI	Negative (0 or 1)	298	96.2	<0.0001
	Positive (≥2)	37	62.2	
LVI	Negative (0)	284	94.6	0.0001
	Positive (≥1)	51	79.7	
TVI	(−)	262	97.3	<0.0001
	(+)	73	74.6	

PL, pleural invasion; BVI, blood vessel invasion; LVI, lymphatic vessel invasion; TVI, tumor vessel invasion.

**Table 6:** **Multivariate analyses of risk factors for recurrence including blood vessel invasion and lymphatic vessel invasion**

**Variable**	**p**	**HR**	**95% CI**
Age	0.185	1.676	0.780–3.600
Sex	0.853	0.930	0.429–2.015
PL	0.016	2.799	1.212–6.464
BVI	0.000	5.669	2.454–13.095
LVI	0.040	2.335	1.039–5.248

PL, pleural invasion; BVI, blood vessel invasion; LVI, lymphatic vessel invasion; HR, hazard ratio; CI, confidence interval.

**Table 7:** **Multivariate analyses of risk factors for recurrence including tumor vessel invasion**

	**p**	**HR**	**95% CI**
Age	0.114	1.851	0.863–3.969
Sex	0.886	0.945	0.438–2.039
PL	0.008	3.015	1.333–6.821
TVI	0.000	6.946	2.980–16.189

PL, pleural invasion; TVI, tumor vessel invasion; HR, hazard ratio; CI, confidence interval.

**Figure 4:**
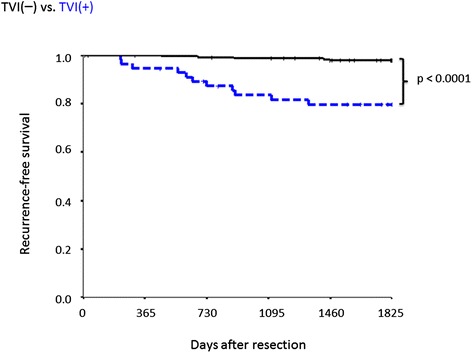
**Recurrence-free survival for patients without pleural invasion according to tumor vessel invasion (TVI).** Comparison between TVI(+) (n = 56) and TVI(−) (n = 243) cases.

The 5-year RFS rates were 94.5%, 74.6%, 96.2%, 62.2%, 94.6%, and 79.7% for the PL(−), PL(+), BVI(−), BVI(+), LVI(−), and LVI(+) groups, respectively (Table 5). Recurrence rates were compared between patients with and without PL. Of 299 PL(−) patients with NSCLC lesions ≤3 cm in diameter, 56 were TVI(+), and 11 of these (19.6%) experienced recurrence; of 243 TVI(−) patients, 7 (2.9%) displayed recurrence. Thus, a significant difference was observed between the two groups (p <0.0001; Figure 4).

### Relationships between vascular invasion, and p-Stages IA and IB

**Figure 5:**
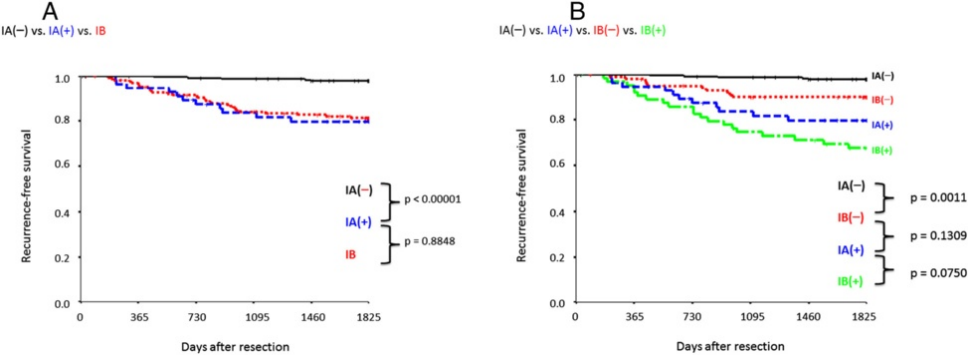
**Recurrence-free survival according to p-Stage and presence of tumor vessel invasion (TVI). A)** Comparison of IA(−) (n = 243), IA(+) (n = 56), and IB (n = 168). **B)** Comparison of IA(−) (n = 243), IA(+) (n = 56), IB(−) (n = 103), and IB(+) (n = 65). Five-year recurrence-free survival rate (%): IA(−), 97.9%; IA(+), 79.7%; IB, 81.3%; IB(−), 81.3%; and IB(+), 67.7%.

Patients with p-Stage IA disease were divided into TVI(+) (IA(+) group) and TVI(−) groups (IA(−) group), and compared with 168 p-Stage IB patients who underwent surgery at approximately the same time (including PL(+) patients with p-Stage IB NSCLC lesions ≤3 cm in diameter; IB group). When the IA(+) and IA(−) groups were compared with the IB groups, there was no significant difference in survival for IA(+) (p = 0.8848), in contrast, survival was significantly different for the IA(−) group (p < 0.00001; Figure 5A).

Similarly, patients with p-Stage IB disease were divided into TVI(+) (IB(+) group) and TVI(−) (IB(−) group) groups, and compared. A significant difference in survival was observed between the IB(+) and IB(−) groups (p = 0.0004). In addition, whereas no significant difference was observed between the IA(+) and IB(−) groups (p = 0.1309), the IB(−) group displayed better survival than the IA(+) group (Figure 5B). The 5-year RFS rates were 97.9%, 79.7%, 81.3%, 89.9% and 67.7% for the IA(−), IA(+), total IB, IB(−), and IB(+) groups, respectively.

## Discussion

In recent years, vascular invasion has been considered an important prognostic factor for patients with p-Stage I NSCLC without metastasis to the lymph nodes [17-23]. Although it is generally believed that HE staining alone is insufficient for vascular invasion diagnoses, few studies have compared HE staining with immunostaining or other specific staining for vascular invasion. Several vascular markers have been previously used. CD31 and CD34 are reactive with vascular endothelial cells; however, CD31 exhibits cross-reaction with platelets, monocytes, and macrophages, and CD34 displays cross-reaction with hematological stem cells and mesenchymal cells. We decided to use EvG staining and podoplanin immunostaining because the former illustrates the blood vessels and clarifies vascular destruction by tumor cells. Podoplanin depicts lymphatic endothelium in the mesenchyme only. Given the difficulty in performing EvG and D2-40 staining of the entire tumor section for all patients in a diagnostic setting, we used the largest tumor sections to ensure realistic representative values (1–5 slide glasses per patient; mean, 1.5 slide glasses per patient in the current study).

We demonstrated that HE staining alone is not sufficient to diagnose vascular invasion accurately, by first examining the diagnosis by HE staining alone, and then adding EvG and D2-40 staining. As shown in Tables 2, 3 and 4, although diagnosis of BVI on HE staining alone revealed significant differences in both univariate and multivariate analyses (HR = 3.945; p < 0.0001 and p = 0.002, respectively), it was apparent that this diagnosis had an inferior sensitivity, as the diagnosis was missed in many patients and their risk of recurrence was not accurately diagnosed. Therefore, HE staining alone cannot accurately identify vascular invasion as similar results were obtained for the diagnosis of ly. HE staining is likely to yield false-negative results in patients who have vascular channels that are difficult to recognize by HE staining alone. This can be because of the destruction of the existing vascular lumen structure, small gaps between the vascular wall and tumor cells, and extremely dense tumors with solid components which results in an indistinct vascular lumen. In contrast, possible reasons for false-positive results following HE staining include a tumor in a prominently thickened alveolar wall owing to advanced fibrosis, easily misidentified as blood vessels, and the presence of tumor cells in gaps created as artifacts during sample preparation. The diagnosis of vascular invasion on HE staining alone may be dependent on the skills of the observer. Although diagnosis by more than one observer may increase accuracy, the addition of EvG and D2-40 staining enables both experienced evaluators and other observers to diagnose vascular invasion readily, and is extremely important for a more accurate diagnosis and assessment of recurrence factors.

Currently, vascular invasion is often assessed using vague qualitative expressions such as “present”, “slight”, and “moderate”. Therefore, we assessed whether there was a significant difference in evaluation of vascular invasion by counting the number of invaded blood vessels on the entire surface of the largest tumor section. A significant difference was noted in the initial comparison between v(+) and v(−), probably because of the predominance of patients with ≥2 v. The most important aspect of the diagnosis of v is not whether it is present or absent, but instead, it is the presence of ≥2 v in the largest tumor section, i.e., the “degree” of v at a given level (assessment considering the BVI). In the diagnosis of ly, a significant difference was observed between the 0 ly and 1 ly groups (p <0.0001), with no further significant difference observed between the 1 ly and ≥2 ly groups. These findings indicate the importance of “presence” in ly diagnosis (assessment considering the LVI).

Many authors have reported that the presence of vascular invasion was a significant prognostic factor [10,15-17,19-23]; however, these studies only included patients with a high degree of vascular invasion. Recently, Kaseda et al. reported that the presence, but not the extent of invasion, was a significant prognostic factor; however, they used glass slides that were previously stained using two different elastic fiber staining methods, which might have resulted in discoloration and have affected the certainty of the evaluation [24]. The assessment should be performed under controlled conditions wherever possible, as applied in this study. Some authors studied other aspects of vascular invasion. Brechot et al. found no prognostic significance when they compared invasion of arteries and veins [14]. Saijo et al. reported the prognostic significance of the location of lymphatic vessels (inside vs. outside the tumor nodule) [18]. The location of vascular invasion might be an important prognostic factor, and further evaluation is warranted.

Image analysis of NSCLC microvasculature is one of the methods used for vessel evaluation. Kayser et al. measured tumor vascularization with an anti-CD34 antibody and quantitative image analysis [25,26]. Szöke et al. reported that an increase in microvessel volume fraction pointed to a poorer survival rate [27]. Generalization of image analysis on specimens would ensure reproducible and accurate vessel evaluation in daily practice.

The current TNM classification states that T1 should be upstaged to T2 when PL is identified. However, the presence of vascular invasion is still not a factor considered in the p-Stage classification despite it being believed to be significantly associated with recurrence [17-23]. Accordingly, we assessed vascular invasion by multivariate analyses and found that PL, BVI, and LVI were independent factors for recurrence. In addition, the prognostic value of BVI (HR = 5.669) was superior to that of PL (HR = 2.799), indicating that BVI is more strongly associated with recurrence than PL. As there is no criterion for changing the p-Stage classification according to vascular invasion, patients with NSCLC lesions ≤3 cm in diameter without PL are all diagnosed with p-Stage IA disease. However, given that the classification of such patients according to BVI results in significantly different recurrence rates, the present classification of these patients in the same p-Stage could be improved.

Consequently, we compared p-Stage IA patients with 168 p-Stage IB patients who underwent resection using the same operative procedure at approximately the same time. Figure 5A shows that the survival of the IA(−) group was significantly different from that of both the IA(+) and IB groups, whereas no difference in survival was noted between the IA(+) and IB groups. However, the IB(−) group tended to have better survival than the IA(+) group (Figure 5B). Although postoperative chemotherapy is currently recommended for p-Stage IB patients [1-6], p-Stage IA patients with TVI are only monitored and do not receive treatment postoperatively. The administration of postoperative treatment equivalent to that given to p-Stage IB patients could lead to an improved outcome for p-Stage IA patients u.

## Conclusions

TVI is an important factor that may increase the recurrence rate; a similar effect by PL was considered significant when the TNM classification system was revised. Although HE staining alone is not sufficient to diagnose vascular invasion, the addition of specific staining and immunostaining may increase objectivity. In the diagnosis of v in particular, the extent of invasion (“degree” of BVI) is more strongly associated with recurrence than PL. In the future, this variable should be considered to determine whether p-Stage IA TVI(+) patients should be administered postoperative chemotherapy similar to that received by patients with p-Stage IB disease, given that they have a comparable risk of recurrence. A further prospective study with a larger cohort is needed to clarify the efficacy of postoperative treatment for p-Stage IA patients with TVI.
